# CHD7 Regulates Osteogenic Differentiation of Human Dental Follicle Cells via PTH1R Signaling

**DOI:** 10.1155/2020/8882857

**Published:** 2020-09-21

**Authors:** Caojie Liu, Qiwen Li, Qingyue Xiao, Ping Gong, Ning Kang

**Affiliations:** West China Hospital of Stomatology, Sichuan University, Chengdu, Sichuan Province, China

## Abstract

Chromodomain helicase DNA-binding protein 7 (CHD7) is an ATP-dependent chromatin remodeling enzyme, functioning as chromatin reader to conduct epigenetic modification. Its effect on osteogenic differentiation of human dental follicle cells (hDFCs) remains unclear. Here, we show the CHD7 expression increases with osteogenic differentiation. The knockdown of *CHD7* impairs the osteogenic ability of hDFCs, characterized by reduced alkaline phosphatase activity and mineralization, and the decreased expression of osteogenesis-related genes. Conversely, the *CHD7* overexpression enhances the osteogenic differentiation of hDFCs. Mechanically, RNA-seq analyses revealed the downregulated enrichment of PTH (parathyroid hormone)/PTH1R (parathyroid hormone receptor-1) signaling pathway after *CHD7* knockdown. We found the expression of PTH1R positively correlates with CHD7. Importantly, the overexpression of *PTH1R* in *CHD7*-knockdown hDFCs partially rescued the impaired osteogenic differentiation. Our research demonstrates that CHD7 regulates the osteogenic differentiation of hDFCs by regulating the transcription of PTH1R.

## 1. Introduction

Originated from ectomesenchymal cranial neural crest cells, dental follicle is a loose connective tissue surrounding the cervical margin of unerupted tooth [1]. The dental follicle could give rise to alveolar bone, cementum, periodontal ligament, gingiva, and other periodontal supporting tissues during the process of tooth germ development [2].

hDFCs are abundant in adolescent patients and easy to obtain [3]. The application of hDFCs in clinic also faces little ethical issue [2]. More importantly, hDFCs are capable of the multilineage differentiation into osteoblasts, fibroblasts, adipocytes, and neurons under different induction cues [4]. Therefore, hDFCs are ideal source for periodontal tissue engineering and regenerative medicine [2, 5].

According to the previous researches, a series of transcription factors or signaling pathways participate in the osteogenetic differentiation of hDFCs, e.g., BMP2, DLX3, NOTCH, Hedgehog, and WNT signaling pathway [6]. As for the gene expression, histone modifications and chromatin remodeling are critical regulatory factors [7].

The chromodomain helicase DNA-binding (CHD) protein superfamily is a typical kind of ATP-dependent chromatin remodeling enzymes of eukaryotic organisms [8]. In the chromatin reading state, CHD protein could disrupt the tissue-DNA interaction by translocating the nucleosomes along the DNA strand [9]. With specific function for active or suppressive histone markers, CHD family are critical for the normal gene expression and maintenance of chromatin dynamic structures [10]. Therefore, the CHD superfamily is essential to stem cell maintenance and proliferation, as well as the regulation of cell fate and differentiation [11].

According to the existed researches, one of the major functions of chromodomain is binding to the methylated histone residues, and correspondingly, the CHD superfamily contains the special methy1-binding cages, which could promote interaction with the histone H3 methylated at lysine 4 (H3K4me) [12].

CHD7 is one of the most studied members of the CHD family because of its extensive and important role in organ development [10]. As a kind of ATP-dependent chromatin remodeling enzymes, CHD7 could regulate the position of nucleosomes and change the accessibility of DNA [13]. Through ChIP, Schnetz et al. found that the recruitment of CHD7 was closely associated with histone modifications, especially H3K4me [14]. Reported previously, CHD7 colocates with H3K4me1 in the enhancer region and with H3K4me3 in the transcription start site [14].

The mutation of CHD7 causes CHARGE syndrome, a developmental disorder that involves multiple organ system defects, including coloboma of the eye, heart defects, atresia of the choanae, retarded growth, genital anomalies, ear malformations, and deafness [15]. The acronym for the six main symptom was defined as CHARGE syndrome by Pagon et al. in 1981 [16].

Previous clinical studies have researched patients with CHARGE syndrome, and most phenotype descriptions were about neural development and neurological disease [17]. Phenotype on bone development was also reported and reviewed in several researches [16]. According to the physical and computed tomography examination, square-shaped face, semicircular canal anomaly, temporal bone abnormality and reduction in bone mineralization can be observed in patients with CHARGE syndrome [18].

We have previously shown that CHD7 plays an important role in osteogenic differentiation of human bone mesenchymal stem cells (hBMSCs) [19]. CHD7 promotes the osteogenic differentiation of hBMSCs by binding to SP7 enhancer and interacting with SMAD1 [19].

In our present work, we focused on the effect of CHD7 on osteogentic differentiation of hDFCs and the downstream signal mechanism. Our results demonstrate that CHD7 promotes osteogenesis of hDFCs via PTH/PTH1R signaling pathway.

## 2. Methods and Materials

### 2.1. Human Dental Follicle Immunohistochemistry (IHC) Staining

Human dental follicle was obtained from the unerupted third molar with undeveloped root. Patients aged 12-16 years undergoing the third molar extraction in West China Hospital of Stomatology, Sichuan University, would meet the inclusion criteria. Patients with history of systemic disease, or undergoing maxillofacial surgery, whole body or partial radiotherapy, chemotherapy, periodontitis, oral mucosal disease, or smoking were excluded [20]. All the procedures are approved by the Institutional Review Board and the informed consent of patients.

The human dental follicle was fixed in 4% polyoxymethuylene for 24 hours before sectioning (5 *μ*m). Slides were incubated in sodium citrate antigen retrieval solution at 100°C for 10 min and then incubated with rabbit anti-CHD7 antibody (Sigma, 1: 200) [21].

### 2.2. Human Dental Follicle Cell Culture

After extraction, the dental follicle was instantly immersed into the phosphate buffer saline (PBS, Gibco) with 1×penicillin-streptomycin (Liquid, Gibco), i.e., PBS with 100 units/mL penicillin and 100 *μ*g/mL streptomycin. The dental follicle was cut into size of 1 mm^3^ pieces and digested with PBS with type I collagenase (3 mg/ml, Gibco) and dispase (3 mg/ml, Gibco) for 1 hour in 37°C water bath with agitation [20].

The digested tissue suspension was cultured in 21cm^2^ petri dish in alpha minimum Eagle's medium (*α*-MEM, HyClone) with 20% fetal bovine serum (FBS, Gibco) and 1×penicillin-streptomycin. Incubated at 37°C with 5% CO_2_, the culture medium was changed every 2 days. When cell confluence rate reached 80%, hDFCs were passaged and subcultured in *α*-MEM with 10% FBS and 1×penicillin-streptomycin. hDFCs at passage 3 were applied for the subsequent research [20].

For the osteogenic induction, hDFCs were cultured with osteogenic medium supplemented with 50 *μ*M ascorbic acid (Sigma), 10 nM dexamethasone (Sigma), and 10 mM *β*-glycerophosphate (Sigma) [22].

### 2.3. CHD7 Knockdown

Cells with ~50% confluence were suitable for the siRNA-mediated knockdown. We obtained targeting control and CHD7 siRNA from Shanghai Sangon Biotech Co. (China). According to the manufacturer's protocol, Lipofectamine® RNAiMAX (Invitrogen) and siRNA were, respectively, added into Opti-MEM (Gibco). The transfection system was mixed and incubated at room temperature in dark for 30 minutes. The medium for hDFCs was changed into *α*-MEM with 10% FBS without antibiotics, then the transfection system with CHD7-siRNA or control-siRNA was added into the corresponding hDFC groups. After 24-hour incubation, the knockdown efficiency was detected via quantitative reverse transcription polymerase chain reaction (qRT-PCR). The sequence of CHD7-siRNA is CCATGAAAGCAATGAGTAA, and of control-siRNA is TACAACAGCCACAACGTCTAT [19].

### 2.4. Lentivirus and Adenovirus-Mediated Overexpression

For the lentivirus-mediated overexpression, lentivirus vectors Ubi-MSC-3FLAG-SV40-EGFP-IRES-puromycin expressing CHD7 or blank were purchased from Shanghai Genechem Co. (China). HitransG/A (Shanghai Genechem) was introduced to enhance the infection efficiency [20].

For the adenovirus-mediated overexpression, adenovirus particles pAV [Exp]-CMV > EGFP expressing PTH1R or blank were purchased from Cyagen (US Inc.) [21].

When reached 20-30% confluence, hDFCs were infected with lentivirus vectors or adenovirus particles at MOI = 20. After 72-hour incubation, 70-80% of the hDFC expressed green fluorescence. The overexpression efficiency was detected via qRT-PCR and Western blot after 2.5 *μ*g/mL puromycin (Sigma) selection [20].

### 2.5. RNA Extraction and qRT-PCR

Total RNA was isolated with TRIzol Reagent (Invitrogen) 3 days and 7 days after the osteogenic induction [20]. RNA was reverse transcribed via a PrimeScript RT reagent Kit (Takara) [23].

Quantitative PCR was performed using SYBR Premix Ex Taq (Takara) and LightCycler 96 (Roche). The house-keeping gene *GAPDH* was used as the baseline to analyze the bone formation-related gene quantitatively [23].

### 2.6. Total Protein Extraction and Western Blot

The total protein of hDFCs was collected with a protein extraction kit (PE001, Sab-biotech) 7 days after the osteogenic induction [4]. The total protein was heated with SDS-PAGE Sample Loading Buffer (Beyotime, China) at 100°C for 5 minutes [24].

After gel electrophoresis separation, protein was transfered to the PVDF membrane (Millipore) via BIO-RAD Powerpac HC. After antigen blocking, the membranes were incubated in rabbit anti-*α*-tubulin antibody (Sigma, 1: 2500), rabbit anti-CHD7 antibody (Sigma, 1: 1000), and mouse anti-PTH1R antibody (Sigma, 1: 1000) at 4°C overnight. After 1-hour incubation with HRP-labeled Goat Anti-Rabbit IgG or Goat Anti-Mouse IgG (Beyotime, China) at room temperature, the membranes were exposed via ChemiDoc XRS+ (BIO-RAD), to detect the selected protein expression level [20].

### 2.7. Alkaline Phosphatase (ALP) Staining and Quantitative Analysis of ALP Activity

hDFCs were fixed with 4% paraformaldehyde and then stained with a BCIP/NBT Alkaline Phosphatase Color Development Kit (Beyotime, China) after the 7-day osteogenic induction. After 15-minute light-free incubation at room temperature, the reaction was terminated, and images were obtained with Epson Perfection V370 Photo Scanner [20].

BCA Protein Assay Kit (Beyotime, China) and Alkaline Phosphatase Assay Kit (Beyotime, China) were used for quantitative analysis of ALP activity. The curve of BCA was obtained from the absorbance of BCA protein concentration gradient. By reaction with 0.5 mM p-nitrophenyl phosphate, the corresponding ALP activity was calculated from the standard curve of ALP absorbance [20].

### 2.8. Alizarin Red S (ARS) Staining and Quantitative Analysis of Mineralization

After the 3-week osteogenic induction in 24-well plates, hDFCs were fixed with 4% paraformaldehyde and then stained with Alizarin Red S solution (Solarbio, China) at room temperature [25].

To quantify the calcium concentration, the calcium nodules were detained by cetylpyridinium chloride for 15 minutes. The quantitative result was measured by absorbance at 562 nm with Multiskan Sky Microplate Spectrophotometer (Thermo Fisher Scientific), in contrast with the standard calcium absorbance curve [21].

### 2.9. RNA-Sequence

RNA samples of hDFCs, 3 samples in siCTRL group and 3 samples in siCHD7 group, were prepared according to the manufacturer's protocol of a NEBNext Ultra RNA Library Prep Kit for Illumina (USA) [21]. RNA samples were subjected to Illumina HiSeq 2500 (USA). FastQC (v0.11.5) and FASTX toolkit (0.0.13) were used for quality control [21]. On the basis, GO enrichment, KEGG enrichment, heat map, and GSEA analysis were conducted to explore the downstream pathway [21].

### 2.10. Chromatin Immunoprecipitation (ChIP) Assay

According to the manufacturer's protocol of a ChIP assay kit (Beyotime, China), 2 × 106 cells were used in each ChIP reaction [19]. By applying 37% formaldehyde solution, protein and DNA of each sample were crosslinked. After cell harvesting, SDS lysis buffer with protease inhibitor cocktail (Roche) was added. After ultrasonication, centrifugation, and precipitation with beads, the precipitated DNA samples were quantified with specific primers using real-time PCR [21].

### 2.11. Statistical Methods

All data were calculated as the mean ± standard deviation (SD). Statistical difference was calculated via Student's *t* test for independent sample test or one-way ANOVA for multiple comparison. *P* value less than 0.05 was considered statistically significant.

## 3. Results

### 3.1. The High Expression of CHD7 in hDFCs after Osteoinduction

**Figure 1:**
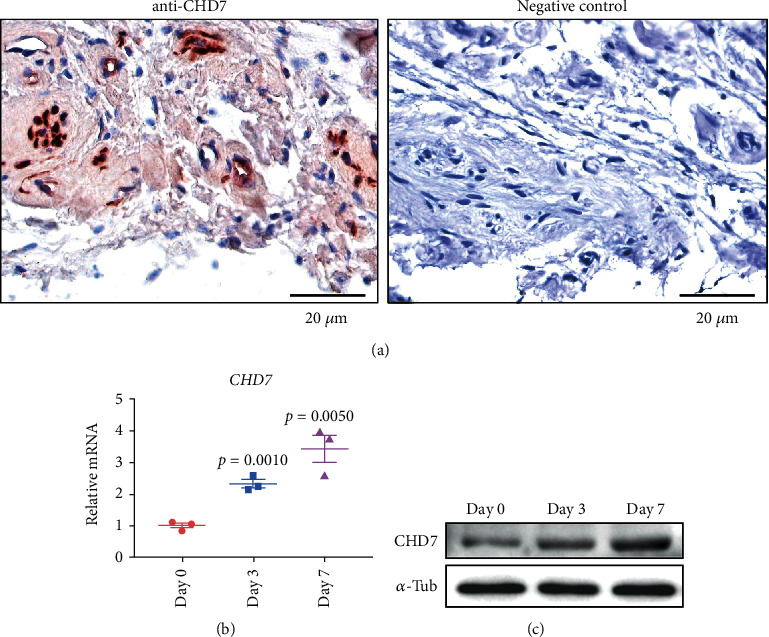
The high expression of CHD7 in hDFCs after osteoinduction. (a) Immunohistochemical staining images unraveled that CHD7 is present in human dental follicle. Scale bar, 20 *μ*m. (b), (c) qRT-PCR and Western blot unraveled that the expression of CHD7 increased after 3-day and 7-day osteoinduction.

We first detected the expression of CHD7 in human dental follicle by IHC staining. Located in nucleus, the high expression of CHD7 can be observed from the slices (Figure 1(a)), which implied that CHD7 might be crucial to the physiological function in dental follicle.

We next conducted the osteogenic induction on hDFCs and analyzed the CHD7 expression changes. As indicated by qRT-PCR, the relative mRNA level of *CHD7* increased after 3-day and 7-day induction (Figure 1(b)). Western blot demonstrated the corresponding trend (Figure 1(c)). These results indicated that CHD7 might be essential to the osteogenic differentiation of hDFCs.

### 3.2. The Knockdown of *CHD7* Decreases Osteogenesis of hDFCs

**Figure 2:**
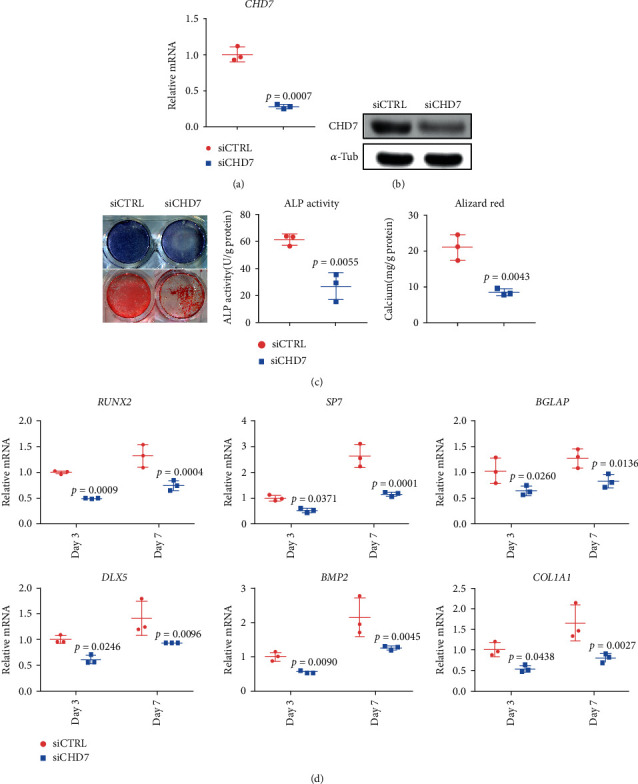
Depletion of *CHD7* decreases osteogenic differentiation of hDFCs. (a), (b) qRT-PCR and Western blot verified the knockdown efficiency of siCHD7. (c) Representative images and quantitative analyses of ALP and ARS staining of hDFCs in the siCHD7 and siCTRL group. (d) qRT-PCR analyses of the expression of *RUNX2*, *SP7*, *BGLAP*, *DLX5*, *BMP2*, and *COL1A1* under osteogenic condition.

To elucidate the role of CHD7 in the osteogenic differentiation of hDFCs, we used siRNA to knockdown *CHD7* in hDFCs. qRT-PCR and WB results verified the efficient knockdown of *CHD7* (Figures 2(a) and 2(b)). 7 days after osteoinduction, the ALP staining demonstrated significant reduction in the *CHD7* knockdown group (Figure 2(c)). The quantitative analysis of ALP activity demonstrated the consistent result (Figure 2(c)). The ARS staining and quantitative analysis of the calcium concentration also confirmed the downtrend of osteogenesis after *CHD7* depletion (Figure 2(c)). Moreover, the expression of osteogenesis-related genes *RUNX2*, *SP7*, *BGLAP*, *DLX5*, *BMP2*, and *COL1A1* was significantly downregulated (Figure 2(d)). The results indicated that the knockdown of *CHD7* reduced the osteogenic differentiation of hDFCs.

### 3.3. The Overexpression of *CHD7* Increases Osteogenesis of hDFCs

**Figure 3:**
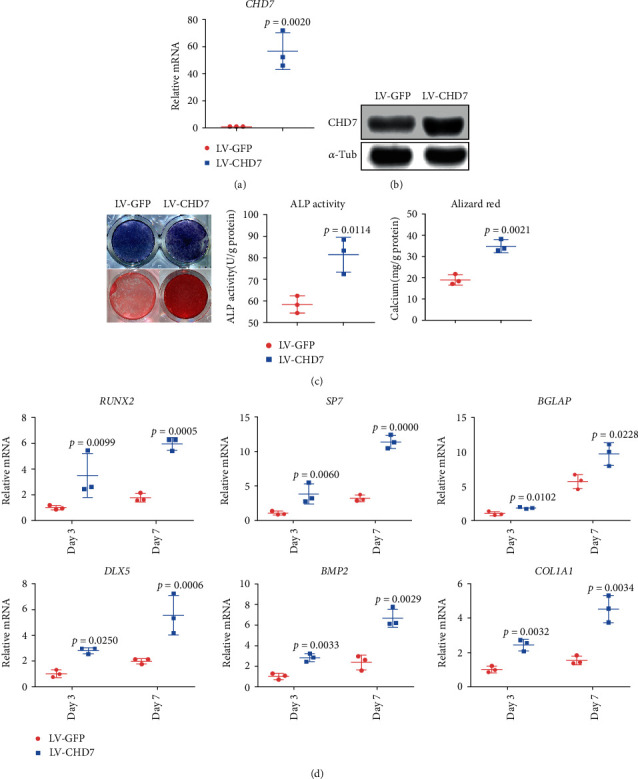
The overexpression of CHD7 promotes osteogenic differentiation of hDFCs. (a), (b) qRT-PCR and Western blot verified the overexpression efficiency of *CHD7*. (c) Representative images and quantitative analyses of ALP and ARS staining of hDFCs in the LV-CHD7 and LV-GFP group. (d) qRT-PCR analyses of the expression of *RUNX2*, *SP7*, *BGLAP*, *DLX5*, *BMP2*, and *COL1A1* under osteogenic condition.

To further elucidate the role of CHD7 in osteogenesis of hDFCs, we infected lentivirus overexpressing *CHD7*, or *GFP* as control, into hDFCs (Figures 3(a) and 3(b)). The overexpression of *CHD7* significantly promoted the osteogenesis of hDFCs, characterized by increased ALP activity, mineralization (Figure 3(c)), and expression of osteogenesis-related genes (Figure 3(d)).

### 3.4. The PTH-Related Pathway Is Downregulated after *CHD7* Depletion

**Figure 4:**
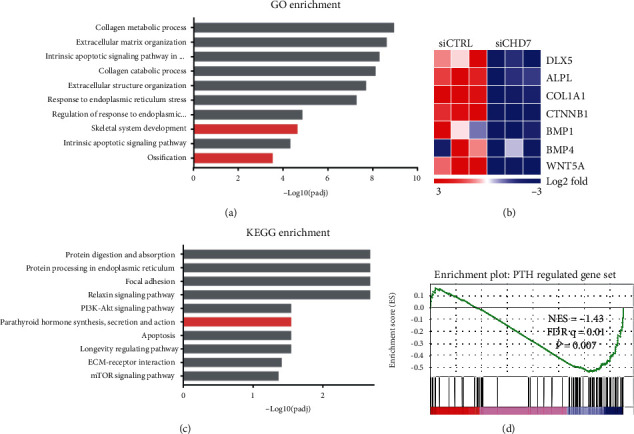
RNA-seq revealed the downregulated enrichment of the PTH-related pathway after CHD7 depletion. (a) GO enrichment unraveled that skeletal system development and ossification were suppressed after *CHD7* depletion. (b) Heatmap of representative osteogenesis associated genes. (c) KEGG enrichment unraveled that the PTH-related pathway was significantly suppressed after *CHD7* depletion. (d) GSEA showed decreased enrichment of PTH-regulated genes in CHD7-deficient hDFCs.

To elucidate the regulatory mechanism of CHD7, we conducted the RNA-seq analysis. GO enrichment showed that the skeletal system development and ossification were suppressed after *CHD7* depletion (Figure 4(a)). Heatmap was generated with recognized osteogenesis-related genes, and most of them were downregulated after CHD7 depletion (Figure 4(b)).

KEGG enrichment identified that the PTH-related pathway was significantly downregulated (Figure 4(c)). According to the previous researches, the PTH/PTH1R signaling pathway is crucial in bone formation and ossification, which is aligned with our phenotype. In order to confirm the downregulation of PTH1R, we conducted the gene set enrichment analysis (GSEA) with the published gene list of the PTH-related pathway [26]. The normalized enrichment score (NES) was -1.43, indicating that the PTH-related pathway was downregulated (*p* = 0.007) (Figure 4(e)). These results implied that the PTH/PTH1R signaling pathway might be a potential target for CHD7.

### 3.5. CHD7 Regulates the Expression of PTH1R

**Figure 5:**
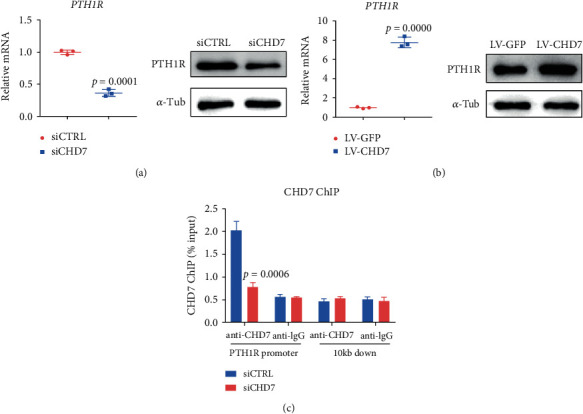
CHD7 regulates the expression of PTH1R. (a) qRT-PCR and Western blot of the PTH1R expression after *CHD7* depletion. (b) qRT-PCR and Western blot of the PTH1R expression after the *CHD7* overexpression. (c) Anti-CHD7 ChIP assay. CHD7 can bound to the promoter region of PTH1R, and the ChIP signaling was significantly suppressed after *CHD7* depletion.

To figure out the regulatory role of CHD7 on PTH1R, we first analyzed the gene and protein expression pattern of PTH1R and CHD7. We found that the mRNA and protein level of PTH1R decreased after the *CHD7* knockdown (Figure 5(a)). Correspondingly, the mRNA and protein level of PTH1R increased after the *CHD7* overexpression (Figure 5(b)). To directly clarify the regulation of CHD7 on PTH1R, we conducted an anti-CHD7 ChIP assay. The result showed that CHD7 can bind to the promoter region of PTH1R, and the ChIP signaling was significantly suppressed after the *CHD7* knockdown (Figure 5(c)).

### 3.6. The Overexpression of *PTH1R* Partially Rescues the Osteogenesis of *CHD7*-Defected hDFCs

**Figure 6:**
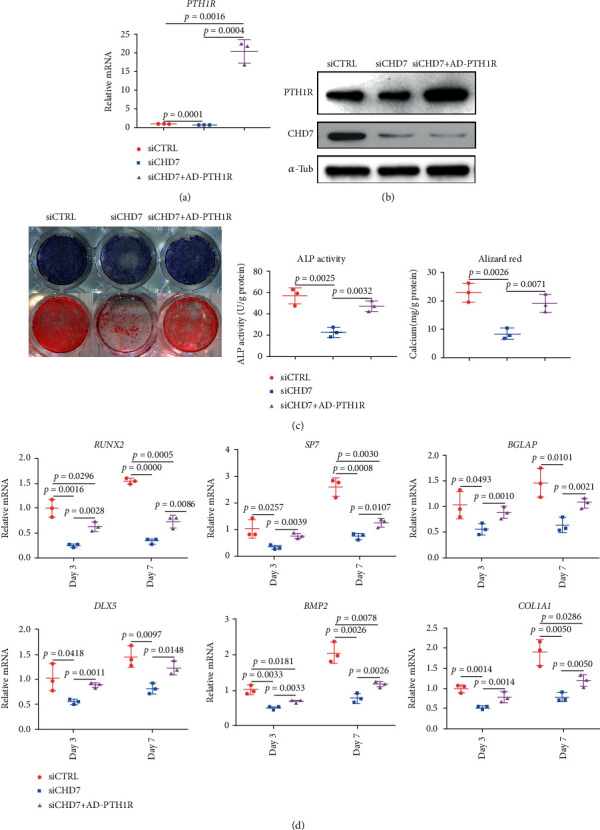
The overexpression of *PTH1R* partially rescues the osteogenic differentiation of CHD7-deficiency hDFCs. (a), (b) qRT-PCR and Western blot verified the overexpression efficiency of *PTH1R*. (c) Representative images and quantitative analyses of ALP and ARS staining of hDFCs in the siCTRL, siCHD7, and rescue group. (d) qRT-PCR analyses of the expression of *RUNX2*, *SP7*, *BGLAP*, *DLX5*, *BMP2*, and *COL1A1* under osteogenic condition.

We next overexpressed *PTH1R* in *CHD7*-knockdown hDFCs (Figures 6(a) and 6(b)). In contrast to *CHD7*-knockdown hDFCs, the overexpression of *PTH1R* significantly promoted the osteogenesis of hDFCs, characterized by increased ALP activity, mineralization (Figure 6(c)), and expression of osteogenesis-related genes (Figure 6(d)). Such results indicated that the overexpression of *PTH1R* could rescue the osteogenic differentiation of *CHD7*-knockdown hDFCs.

## 4. Discussion

The osteogenic differentiation of hDFCs is a complex process, which involves a variety of intracellular and extracellular signaling pathways [27]. As a chromadomain helicase, the chromatin remodeling function of CHD7 is performed by identifying and binding to specific histone modification sites of nucleosomes, using the energy provided by ATP hydrolysis to make chromatin deagglutination and expose DNA and to increase the accessibility of transcription regulatory elements [28].

CHD7 is not only located in nucleoplasm to regulate the transcription of many genes but also in nucleolus to regulate the production of ribosomal RNA [8, 29]. Once intracellular ribosome production is disturbed, protein synthesis will also be seriously affected. Some rapidly proliferating cells in development like neural crest cells are particularly sensitive to such event [30]. According to previous studies, the neural crest abnormality might be the main cause of the corresponding tissue abnormality of CHARGE syndrome [29, 31]. Dental follicle is also originated from ectomesenchymal cranial neural crest cells [2]. This might be an alternative explanation for the osteogenetic dysfunction of hDFCs after the *CHD7* knockdown, which deserves further study.

PTH1R is one of the direct signaling mediators in promoting the osteogenic differentiation of BMSCs [21]. According to our result, CHD7 is vital for the translation of PTH1R during osteogenesis of hDFCs. The *CHD7* knockdown reduces the expression level of PTH1R and impairs the osteogenic function of hDFCs. Subsequently, the *PTH1R* overexpression in the CHD7-knockdown hDFCs partially rescued the impaired osteogenic potential.

The function of the PTH/PTHrP signaling pathway during tooth root formation has been reported by previous researches. The PTH/PTHrP signaling pathway could maintain physiological cell fate of dental follicle mesenchymal progenitor cells to generate functional periodontium and coordinate tooth eruption [24, 32, 33]. During the process of tooth root formation, PTHrP^+^ dental follicle cells could differentiate into cementoblasts on the basis of acellular cementum with periodontal ligament cells and alveolar bone osteoblasts [34].

According to the qRT-PCR results, although the *PTH1R* overexpression level was considerably high, the rescue of hDFC osteogenic differentiation was still partially, rather than entirely. One possible explanation is that there might be other downstream pathways of CHD7 in hDFCs [6]. On the basis of RNA-seq analysis, exploration in the mechanism, and the previous researches, we inferred that PTH1R is vital and might be the major mechanism of CHD7 during tooth development [34]. Besides, according to RNA-seq analysis, the downregulated gene also includes classic osteogenic regulatory pathway, e.g., *WNT5A*, *NFIC*, and *BMP1.* It could be implied that there can also be other important downstream regulators, which could be our further research targets [4].

Besides CHD7, other member in the CHD family also contribute to tooth root development. Previous research has confirmed high and increasing expression of CHD3 in early and middle stage of tooth root formation, especially in Hertwig's epithelial root sheath [35]. Depletion of CHD3 and cDNA microarray analysis suggested that CHD3 might play a positive role in DNA synthesis in Hertwig's epithelial root sheath cells in tooth development, especially tooth root formation [36].

Several limitations in our research should be noted to provide ideas for the further study. First, for the lack of *CHD7* knockout mice, our research could not conduct corresponding animal experiment. Phenotype in vivo should be studied in the future to reveal the function and mechanism of CHD7 more comprehensively. Moreover, the mechanism of CHD7 regulating the osteogenic differentiation in hDFCs might be associated with the ribosomal RNA production [37]. More studies in molecular genetics and developmental biology could provide more significant evidence.

## Data Availability

All Seq data have been deposited into NCBI database with the identifier GSE154822.
